# Knee arthroplasty can preserve cognitive function in patients with severe knee osteoarthritis

**DOI:** 10.1371/journal.pone.0317707

**Published:** 2025-08-28

**Authors:** Juneyoung Heo, Ji-Hoon Baek, Chang Hyun Nam, Taehyeon Kim, Hye Sun Ahn, Su Chan Lee

**Affiliations:** 1 Joint & Arthritis Research, Department of Neurosurgery, Himchan Hospital, Seoul, Republic of Korea; 2 Joint & Arthritis Research, Department of Orthopaedic Surgery, Himchan Hospital, Seoul, Republic of Korea; Government Law College, INDIA

## Abstract

**Background:**

This study examined how the clinical and radiological severity of knee osteoarthritis (OA) and various treatment methods affect cognitive function.

**Method:**

This prospective study included patients with knee pain or discomfort who visited a single institution between July 2017 and November 2022. Patients with Kellgren–Lawrence (K–L) grade 0 or treatable dementia were excluded from this study, and 88 patients were followed up for an average duration of 2.4 ± 1.3 years. Overall, 55 patients had K–L grade 4, of whom 45 who did not improve after >3 months of conservative treatment underwent knee arthroplasty.

**Results:**

Among patients with K–L grade 4, the decrease in the Mini-Mental State Examination score in patients who underwent surgery was significantly lower than that in other patients who received conservative treatment only.

**Conclusion:**

Knee arthroplasty can reduce the decline in cognitive function in patients with severe knee OA of K-L grade 4 who do not respond to conservative treatment. Even if pain is controlled with conservative treatment, the decline in cognitive function in patients with OA is faster than the age-related decline observed in the general population.

## Introduction

Osteoarthritis (OA) is a chronic, progressive disease that affects the joints and surrounding tissue. Among them, the knee is the most commonly affected joint, with knee OA affecting >10% of men and 13% of women aged >60 years. Knee OA causes pain and decreased function, and the resulting socioeconomic burden has increased over the past few decades and is expected to increase in the future [1,2]. Furthermore, dementia is a progressive neurodegenerative disease that is common among the older population. In total, 55 million patients were affected by this disease worldwide in 2021, and the number is estimated to increase to 150 million by 2050. Dementia is characterized by a progressive loss of cognitive function, which may be caused by Alzheimer’s disease (AD), accounting for the largest proportion of dementia cases, or other factors. Currently, there is no cure for AD, and only treatment to alleviate symptoms is available [3]. Therefore, preventing dementia and managing risk and exacerbation factors are crucial.

OA is not only characterized by the loss of cartilage due to mechanical loading but also affects the tissues surrounding the subchondral bone and joint, inducing changes in tissue architecture, metabolism, and function. Although these changes have not yet been fully elucidated, they occur through various complex mediators, including cytokines, adipokines, and growth factors, which are also used as biomarkers to estimate the extent of disease progression. These mediators may affect the entire body because they are found not only in the tissues around the joint, including the synovium, but also in the serum. Initially, OA was only considered a problem caused by mechanical damage and wear to the local cartridge. However, it is now widely recognized as a local peripheral low-grade inflammation that occurs throughout a joint, including the synovium and subchondral bone. Nevertheless, it remains unclear whether peripheral low-grade inflammation is associated with systemic inflammatory-associated disease [4–6]. Recently, peripheral low-grade inflammation has been suggested as a risk factor for AD in several clinical and animal studies [7–12].

To date, several animal experiments and clinical trials have analyzed the relationship between knee OA and cognitive function. Among them, animal experiments have shown that OA can induce or accelerate the progression of AD [13,14], and large population-based studies conducted in Taiwan and Japan have demonstrated that OA is an independent risk factor for dementia (or mild cognitive impairment [MCI]) [15,16]. Based on previous research findings, we hypothesized that higher severity of knee OA leads to a greater decline in cognitive function and that performing total knee arthroplasty (TKA) for severe knee OA may help reduce the rate of cognitive decline. This prospective study investigated the impact of the clinical and radiological severity of knee OA and treatment methods on cognitive function.

## Materials and methods

The design and protocol of this prospective study were approved by the institutional review board (IRB) of our hospital, which waived the requirement for informed consent (IRB number: 116655-01-202401-02). Data for the presented analyses were accessed on January 25, 2024.

This study involved patients with knee pain or discomfort who visited a single institution between July 2017 and November 2022. All patients underwent history taking, physical examination, and roentgenography of both knees; additional tests such as magnetic resonance imaging were performed when necessary. Patients with Kellgren–Lawrence (K–L) grade 0 on X-ray were excluded from this study. The K–L grading system (graded from 0 to 4) is a radiographic classification system of knee OA based on roentgenography. Patients with knee pain underwent conservative treatments, including nonsteroidal anti-inflammatory drugs (NSAIDs) and physical therapy, for at least 3 months, whereas those with K–L grade 4 whose symptoms did not improve despite treatment underwent TKA.

Simultaneously, the comprehensive patient medical history was confirmed by neurologists and neurosurgeons at the same hospital belonging to the same medical institution. As necessary, the patients were examined in the presence of their family members or guardians. Furthermore, initial and follow-up tests for cognitive function were performed using the Mini-Mental State Examination (MMSE). The MMSE is a tool used solely to evaluate cognitive function and does not address mental disorders or behavior-related aspects. It comprises 30 questions and evaluates overall cognitive function such as orientation, language, attention, visuospatial skills, and memory. The MMSE is widely used to screen dementia cases and evaluate cognitive decline in individuals without dementia. If the MMSE score was ≤ 26, physical and neurological examinations were performed to screen for dementia, and the associated risk factors were evaluated. By performing tests such as brain imaging, cerebrospinal fluid study, laboratory test, and thyroid function test, patients with treatable causes of dementia, such as brain tumor, brain infection, hydrocephalus, hepatic encephalopathy, renal failure, vitamin B1 and B12 deficiencies, and electrolyte imbalance, were excluded from the study. In this study, the diagnosis of dementia was based on the Clinical Practice Guideline for Dementia (Diagnosis and Evaluation) in South Korea [17] and the results of a previous study [18].

Patients who underwent TKA used NSAIDs for 2–3 months after surgery to control pain, after which these medications were discontinued. In contrast, patients who did not undergo surgery and whose symptoms improved with drug treatment continued to receive conservative treatment, including NSAIDs. Furthermore, no separate drug treatments were administered for cognitive decline, and the MMSE was administered approximately once every 6 months to confirm changes in cognitive function such as attention, language, memory, orientation, and visuospatial skills.

In total, 231 patients with knee pain or discomfort visited the hospital. After excluding 143 patients with K–L grade 0 and treatable dementia, a prospective study was conducted in 88 patients, among whom 60 (68%) were female. The average patient age was 80.8 ± 6.9 (range, 63–100) years (Table 1). A negative correlation was observed between age and K–L grade and between age and MMSE score, with Kendall’s tau correlation coefficients of −0.1 and −0.258, respectively. Two patients had a history of cerebrovascular disease, seven had cardiovascular disease, and one had arrhythmia. Among the recruited patients, none had autoimmune diseases (e.g., rheumatoid arthritis and systemic lupus erythematosus) (Table 2). Although a few cases of COVID-19 occurred during the follow-up period, most patients recovered quickly without lasting effects, and these cases were not expected to have influenced the results of this study.

**Table 1:** Demographic characteristics and risk factors that can affect knee OA and cognitive function.

	Final cohort
**Number of patients (n)**	88
**Male:female**	28:60
**Age (years) (mean ± SD)**	80.8 ± 6.9 (range, 63–100)
**Medical history (n)**	59
** Hypertension**	59
** Diabetes mellitus**	21
** Hyperlipidemia**	19
** Stroke**	2
** Cardiovascular disease**	7
** Arrhythmia**	1

SD: standard deviation

**Table 2:** Clinical and radiological patient characteristics.

	Final cohort
**K–L grade**	
** 1 or 2**	28
** 3**	5
** 4**	55
**Knee arthroplasty**	
** No operation**	43
** Unilateral**	12
** Bilateral**	33
**MMSE**	
** Average (mean ± SD)**	21.8 ± 4.6
** Minimum**	16
** Maximum**	29
**Follow-up period (years) (mean ± SD)**	2.4 ± 1.3 (range, 0.5–5.7)

K–L: Kellgren–Lawrence, MMSE: Mini-Mental State Examination, SD: standard deviation

Overall, 55 patients had K–L grade 4, of whom 45 underwent knee arthroplasty because conservative treatment for >3 months was ineffective. Among them, 12 patients underwent unilateral knee arthroplasty and 33 underwent bilateral knee arthroplasty. The MMSE scores of the patient group ranged from 16 to 29 (average, 21.8 ± 4.6) points. The average follow-up period was 2.4 ± 1.3 (range, 0.5–5.7) years.

Statistical analyses were performed using SPSS version 27 (Chicago, IL, USA). Variables were analyzed using the independent *t*-test and one-way analysis of variance (ANOVA). All *p*-values were two-sided, and a *p*-value of <0.05 was considered to indicate statistical significance.

### Sample size

The sample size in the current study was 73, as estimated using G*Power 3.1.9.7. Owing to the absence of any prior similar studies, the estimate was based on pilot data comparing MMSE scores at recruitment and 12-month follow-up, and a 17% difference was observed. The calculation was performed by categorizing the participants into two or three groups and performing an independent t-test or ANOVA, respectively. With a significance level of 0.05, power of 0.8, and a large effect size, the required sample sizes were 52 and 66 for the t-test and ANOVA, respectively. Accounting for a 10% withdrawal rate, a sample size of >73 participants was necessary.

## Results

This study confirmed that the decrease in MMSE scores in patients who underwent knee arthroplasty was significantly lower than that in other patients who received conservative treatment only (Table 3). Furthermore, the higher the K–L grade, the lower the decrease in MMSE scores (Table 4); however, this result was not statistically significant after adjusting for whether surgery was performed. Among the group of patients with K–L grade 4, significant results were obtained when comparing patients who underwent surgery with those who received conservative treatment only. The MMSE scores of patients who underwent surgery decreased by only 0.31 ± 2.25 points during the follow-up period, whereas those of patients who did not undergo surgery decreased by 1.33 ± 1.89 points, showing a greater decrease (Tables 3 and 5).

**Table 3:** Differences in MMSE scores between patients who had knee arthroplasty and those who did not.

	Knee arthroplasty	No operation	*p*-value
**Number of patients**	45	43	
**MMSE (mean ± SD)**	21.4 ± 4.5	22.1 ± 4.8	0.470
**Change in MMSE scores** **(mean ± SD)**	0.31 ± 2.3 decrease	1.48 ± 2.2 decrease	0.020

MMSE: Mini-Mental State Examination, SD: standard deviation

**Table 4:** Differences in MMSE scores according to K–L grades.

	K–L grade 1 or 2	K–L grade 3	K–L grade 4	*p*-value
**Number of patients**	28	5	55	
**MMSE score** **(mean ± SD)**	22.0 ± 4.9	20.6 ± 3.0	21.6 ± 4.6	0.739
**Change in MMSE scores** **(mean ± SD)**	1.7 ± 2.5 decrease	0.2 ± 2.9 decrease	0.4 ± 2.1 decrease	0.030

K–L: Kellgren–Lawrence, MMSE: Mini-Mental State Examination, SD: standard deviation

**Table 5:** Differences in MMSE scores according to K–L grades in the patient group (43 patients) that did not undergo surgery.

	K–L grade 1 or 2	K–L grade 3	K–L grade 4	*p*-value
**Number of patients**	28	5	10	
**MMSE score** **(mean ± SD)**	22.0 ± 4.9	19.7 ± 3.8	23.7 ± 4.3	0.530
**Change in MMSE scores** **(mean ± SD)**	1.69 ± 2.5 decrease	1.66 ± 2.9 decrease	1.12 ± 1.3 decrease	0.560

K–L: Kellgren–Lawrence, MMSE: Mini-Mental State Examination, SD: standard deviation

Even after adjusting for age, hypertension, diabetes mellitus, hyperlipidemia, stroke, cardiovascular disease, and arrhythmia, which are factors that can affect cognitive function in addition to surgery, the result remained unchanged: reduction in MMSE scores remained lower in patients who underwent knee arthroplasty (*p* = 0.021–0.024). As shown in Table 6, the MMSE scores were compared according to medical history (e.g., hypertension, diabetes, and stroke), and the scores were lower in patients with hypertension and cardiovascular disease than in those without. Other medical histories did not affect the initially measured MMSE scores. The change in MMSE scores was corrected in patients who did or did not undergo TKA; however, each medical history showed no effect on the change in MMSE scores. Education level exerted a positive effect on MMSE scores; however, TKA did not affect the change in MMSE scores in patients who underwent or did not undergo surgery (Table 6). The change in MMSE scores in patients aged 70–89 years was not affected by age (Table 7). Among patients who did not undergo TKA, the decrease in MMSE scores in patients aged <69 and >90 years was smaller than that in patients from other age groups; however, the significance was limited by the small sample size. The MMSE scores of patients aged >90 years were considerably lower than those of patients from other age groups; therefore, further reduction in their scores is challenging. Studies have shown a high prevalence of dementia in patients with OA [15,16]; however, the results showed a very weak correlation between the K–L grade and MMSE score.

**Table 6:** Changes in MMSE scores according to factors that may affect cognitive function.

Medical history	n.	MMSE score (mean ± SD)	MMSE scores of patients who underwent knee arthroplasty (mean ± SD)	MMSE scores of patients who did not undergo knee arthroplasty (mean ± SD)
**Hypertension**	59	20.9 ± 5.8	Initial: 20.0 ± 4.7	Initial: 21.7 ± 4.6
			Change: 0.29 ± 1.9 decrease	Change: 1.51 ± 2.3 decrease
**No hypertension**	29	22.1 ± 4.5	Initial: 22.3 ± 3.7	Initial: 21.7 ± 5.2
			Change: 0.43 ± 2.5 decrease	Change: 1.39 ± 1.9 decrease
**Diabetes**	21	21.1 ± 3.1	Initial: 20.9 ± 3.7	Initial: 21.7 ± 2.6
			Change: 0.37 ± 2.1 decrease	Change: 1.41 ± 3.2 decrease
**No diabetes**	67	22.0 ± 4.8	Initial: 21.7 ± 4.9	Initial: 22.7 ± 4.1
			Change: 0.30 ± 2.7 decrease	Change: 1.49 ± 1.8 decrease
**Hyperlipidemia**	19	22.1 ± 4.1	Initial: 22.5 ± 3.1	Initial: 24.1 ± 5.8
			Change: 0.29 ± 1.1 decrease	Change: 1.41 ± 2.1 decrease
**No hyperlipidemia**	69	21.7 ± 5.3	Initial: 21.5 ± 3.1	Initial: 20.1 ± 5.1
			Change: 0.32 ± 2.5 decrease	Change: 1.59 ± 2.3 decrease
**Stroke**	2	20.5 ± 2.12	19	22
			Change: 0	Change: 2 decrease
**No stroke**	86	22.0 ± 4.4	Initial: 21.5 ± 4.7	Initial: 22.7 ± 5.5
			Change: 0.35 ± 2.2 decrease	Change: 1.45 ± 2.3 decrease
**Cardiovascular disease**	7	19.1 ± 5.1	Initial: 19.0 ± 4.1	Initial: 19.3 ± 5.7
			Change: 0.32 ± 3.2 decrease	Change: 1.50 ± 1.8 decrease
**No cardiovascular disease**	81	22.2 ± 3.9	Initial: 23.1 ± 3.7	Initial: 22.7 ± 4.0
			Change: 0.27 ± 1.9 decrease	Change: 1.43 ± 3.3 decrease
**Arrhythmia**	1	18	18	N/A
			Change: 1 increase	N/A
**No arrhythmia**	87	22.0 ± 4.3	Initial: 22.0 ± 4.1	Initial: 22.1 ± 4.8
			Change: 0.33 ± 1.8 decrease	Change: 1.48 ± 2.2 decrease
**Highest level of education**				
**Elementary school and below**	51	20.3 ± 2.8	Initial: 21.0 ± 3.1	Initial: 19.7 ± 5.8
			Change: 0.33 ± 1.8 decrease	Change: 1.49 ± 1.2 decrease
**Middle or high school**	31	21.6 ± 5.7	Initial: 21.4 ± 4.1	Initial: 20.9 ± 5.9
			Change: 0.27 ± 3.5 decrease	Change: 1.43 ± 3.5 decrease
**University or higher**	6	25 ± 3.4	Initial: 25.0 ± 2.0	Initial: 25.0 ± 3.1
			Change: 0.30 ± 2.1 decrease	Change: 1.50 ± 2.5 decrease

MMSE, Mini-Mental State Examination; SD, standard deviation

**Table 7:** Changes in MMSE scores by age group.

Age (years)	n	MMSE (mean ± SD)	MMSE scores of patients who underwent knee arthroplasty (mean ± SD)	MMSE scores of patients who did not undergo knee arthroplasty (mean ± SD)
**<69**	8	26 ± 2.55	Initial: 27.0 ± 1.0	Initial: 25.5 ± 3.2
			Change: 0.5 ± 2.8 decrease	Change: 0.72 ± 3.2 decrease
**70–79**	19	22 ± 4.90	Initial: 20.5 ± 3.3	Initial: 24.3 ± 2.1
			Change: 0.4 ± 1.1 decrease	Change: 1.52 ± 3.5 decrease
**80–89**	57	21 ± 4.39	Initial: 22.0 ± 1.7	Initial: 20.0 ± 3.1
			Change: 0.22 ± 3.1 decrease	Change: 1.47 ± 1.9 decrease
**>90**	4	18 ± 6.02	Initial: 14	Initial: 19.0 ± 6.8
			Change: None	Change: 0.66 ± 0.57 decrease

MMSE, Mini-Mental State Examination; SD, standard deviation

## Discussion

MMSE scores are substantially influenced by age and education level, and the average MMSE score gradually decreases as age increases. Although differences occur depending on age and education level, the MMSE score of individuals aged 70–74 years is 0.4–1.0 higher than that of individuals aged 75–79 years, and the decline in MMSE scores increases with advanced age [19–21]. In the current study, the average age of the patient group was 80.8 years, and the average follow-up period was 2.38 years. Therefore, the decrease of 0.31 points in MMSE scores during the follow-up period in patients who underwent surgery can be considered as a natural decrease with aging. The greater decrease in MMSE scores in patients who did not undergo surgery can be interpreted as the effect of knee OA on the decline in cognitive function during the follow-up period.

In summary, knee arthroplasty surgery can help reduce the decline in cognitive function in patients with severe knee OA who show K–L grade 4 findings and are not responsive to conservative treatments. Knee arthroplasty is an effective treatment that can eliminate inflammation not only in the knee cartilage but also in the surrounding soft tissues and can be assumed to be effective in removing factors that cause or worsen dementia in patients with advanced knee OA. Furthermore, even if pain is controlled with conservative treatment, the decline in cognitive function in patients with OA is faster than that in the general population, indicating that OA can further worsen cognitive function.

Knee OA causes not only pain but also limitations in daily activities in 43% of patients [22]. Physical activity limitations due to pain increase not only the risk of obesity and cardiovascular diseases [23] but also the likelihood of developing depressive symptoms. Moreover, they are associated with the possibility of suicidal impulses [24]. Considering this evidence, it can be assumed that knee OA has certain effects on mental health. Furthermore, studies conducted in Taiwan and Japan have shown that patients with knee OA have reduced cognitive function or a high prevalence of dementia [15,16,25]. These studies have considered that the effect of knee OA on cognitive function was an indirect effect caused by pain and decreased activity, which can affect cognitive function [26,27].

However, studies have recently hypothesized that knee OA has a direct effect on dementia. Based on studies on the pathophysiology of knee OA by systemic inflammation, interleukin-1 (IL-1) and tumor necrosis factor-alpha (TNF-α), which are the most important proinflammatory cytokines in the occurrence of knee OA, may worsen AD [15,28]. Moreover, animal experiments conducted in 2011 and 2023 have shown that peripheral inflammation caused by knee OA causes neuroinflammation, increasing beta-amyloid deposition and neuronal loss [13,14]. The current study showed that dementia may worsen in patients with knee OA, suggesting that knee OA has a direct effect on the development or worsening of dementia.

The impact of systemic inflammation on dementia has already been shown in several studies; moreover, peripheral inflammation has been suggested as a risk factor for AD [7,9,10]. Studies have shown that peripheral inflammation leads to neuroinflammation, a chronic response caused by microglia and other immune cells in the central nervous system that is associated with neurodegenerative diseases [29]. Peripheral inflammation increases the transcription of inflammatory cytokines [30]. Among them, IL-1β and TNF-α can pass through the blood–brain barrier (BBB) damaged by neuroinflammation, thereby causing neurodegeneration. Cytokines that pass through the BBB play an important role in neurodegeneration by maintaining the sustained activated state of microglia and other glial cells [29]. Among them, IL-1β induces the production of β-amyloid precursor protein and increases amyloid plaque deposition in AD brains [13,14,31]. Furthermore, animal experiments have proven that β-amyloid generated in this manner accumulates in the hippocampus and cerebral cortex, which is associated with cognitive impairment [32].

In this study, among comorbidities such as hypertension and diabetes, hypertension only affected the preoperative MMSE score and not the change in the MMSE score during the follow-up period (Table 6). However, this study evaluated cognitive function with MMSE. Comorbidities that affect frontal lobe/executive function may not be accurately evaluated. For example, hypertension can affect cognitive function by causing a silent cerebral infarct [33], and if the affected area is the frontal lobe, the MMSE score may be higher than the actual cognitive function. Diabetes-related dementia greatly affects executive function and reduces word recall less [34]; thus, patients with diabetes can benefit from the MMSE score. Therefore, the generalizability of the results of this study is limited, and research using a tool that can better measure the frontal lobe/executive function is warranted.

The MMSE is one of the most widely used tools for screening dementia. It is often used in clinical settings to measure cognitive changes in older individuals over time. It can be administered easily within a short period and has minimal practice effects, allowing for the observation of changes over time via repeated measurements during the disease course. However, several studies have indicated that the MMSE does not sufficiently assess frontal lobe/executive function. Among the cognitive domains, memory and frontal lobe function showed the greatest impairment in the false-negative group. Changes in MMSE scores may result from measurement errors, regression to the mean, practice effects, and normal aging. In a previous study, a change of 2–4 points or more was considered significant at intervals of 1.5–5 years [35,36]. In the present study, MMSE was performed once every 6 months, which may have had a slight practice effect. In addition, regression to the mean may have positively affected MMSE scores. Nevertheless, the decrease in MMSE scores in the non-TKA group was considered significant. The present study focused only on the presence or absence of changes in cognitive function rather than the accurate diagnosis of cognitive function while treating patients with severe knee OA. Therefore, only MMSE was performed. In future studies, the Montreal Cognitive Assessment (MoCA), a screening tool for MCI, can be used to simply evaluate cognitive function. In addition, neuropsychological assessment batteries such as the Consortium to Establish a Registry for Alzheimer’s Disease (CERAD), Seoul Neuropsychological Screening Battery (SNSB), and Alzheimer’s Disease Assessment Scale, which can measure overall cognitive function, may be used to evaluate specific areas of cognitive function that are affected. Several studies have shown that MoCA is more sensitive in assessing executive function and visuospatial ability [37,38] and more effective in detecting mild cognitive impairment (MCI) than MMSE [39]. Several neuropsychological assessment batteries, such as CERAD and SNSB, have proven useful for assessing individual cognitive function [40–42]. The MMSE and MoCA tests can be easily administered and are not associated with increased cost and time; however, they have limited accuracy in assessing cognitive function. Although neuropsychological assessment batteries can accurately measure cognitive function and do not have the disadvantages of measurement error and practice effect, they are time-consuming and costly, which may render research difficult. In particular, administering CERAD or SNSB every 6 months may cause problems with staff availability and budget limitations, and solving problems that last for about an hour may be unpleasant for some participants.

A study conducted in Taiwan [15] had the advantage of having a large number of patients because it was based on the Taiwan longitudinal health insurance database 2005 (LHID 2005). However, the criteria for diagnosing dementia and OA in patients included in the study were unclear, and the study assessed OA of all joints rather than knee OA only. A Japanese study [16] targeted patients with MCI who had MMSE scores of ≤23 but did not mention whether brain imaging and laboratory tests were performed. Both studies have the advantage of being population-based, large-scale studies; however, they have limitations such as ambiguity in diagnostic criteria and inability to determine changes according to treatment. These two studies have shown an epidemiological association between OA and dementia; however, they showed limitations in temporal precedence and causal relationships. Our study is the first to prospectively demonstrate the effects of knee OA treatment on cognitive function. Although existing cross-sectional studies have demonstrated a relationship between knee OA and dementia, our study showed how knee OA treatment affects cognitive decline. As this study was conducted at a single institution, the generalizability of the results may be limited.

In conclusion, for patients with severe knee OA of K–L grade 4 who do not respond to conservative treatment, knee arthroplasty surgery can reduce the decline in cognitive function. Even if pain is controlled with conservative treatment, the decline in cognitive function in patients with OA is faster than that in the general population, suggesting that OA worsens dementia.

### Ethics approvavl and consent to participate

This study was approved by the Institutional Review Board of Himchan hospital (IRB number: 116655-01-202401-02). The requirement for informed consent was waived because of the retrospective nature of this study.
